# Association Between Baseline Neutrophil-to-Lymphocyte Ratio and Short-Term Urinary Quality of Life During BCG Induction in Male Patients with Non-Muscle-Invasive Bladder Cancer: A Prospective Observational Study

**DOI:** 10.3390/jcm14196908

**Published:** 2025-09-29

**Authors:** Lorenzo Spirito, Simone Tammaro, Paola Coppola, Celeste Manfredi, Lorenzo Romano, Carmine Sciorio, Antonio Di Girolamo, Luigi Napolitano, Francesco Bottone, Carmelo Quattrone, Vittorio Imperatore, Ferdinando Fusco, Davide Arcaniolo, Marco De Sio

**Affiliations:** 1 Urology Unit, Department of Woman, Child and General and Specialized Surgery, University of Campania “Luigi Vanvitelli”, 80131 Naples, Italy; lorenzospirito@msn.com (L.S.); simone.tammaro95@gmail.com (S.T.); coppola997@gmail.com (P.C.); lorenzo.romano@unicampania.it (L.R.); bottonefrancesco@yahoo.it (F.B.); carmeloquattrone@hotmail.it (C.Q.); ferdinando-fusco@libero.it (F.F.); davide.arcaniolo@gmail.com (D.A.); marco.desio@unicampania.it (M.D.S.); 2 Urology Unit, “A. Manzoni” Hospital, 23900 Lecco, Italy; carmine.sciorio@gmail.com; 3 Urology Unit, AORN San Giuseppe Moscati, 83100 Avellino, Italy; antonio.digirolamo@hotmail.it (A.D.G.); v.imperatore1@gmail.com (V.I.); 4 Urology Unit, Department of Neurosciences, Reproductive Sciences and Odontostomatology, University of Naples “Federico II”, 80131 Naples, Italy; dr.luiginapolitano@gmail.com

**Keywords:** BCG, bladder cancer, instillation, intravesical, neutrophil-to-lymphocyte ratio

## Abstract

**Background/Objectives:** Intravesical Bacillus Calmette–Guérin (BCG) is the standard adjuvant treatment for high-risk non-muscle-invasive bladder cancer (NMIBC), but treatment-related urinary toxicity may compromise quality of life (QoL) and adherence. The neutrophil-to-lymphocyte ratio (NLR), a marker of systemic inflammation, has been linked to oncologic outcomes in bladder cancer, but its association with urinary symptom burden during BCG therapy remains unclear. We aimed to assess whether baseline NLR is associated with early deterioration in urinary symptoms and urinary QoL following BCG induction. **Methods:** This prospective study included male patients with NMIBC treated with intravesical BCG. Baseline demographics, comorbidities, laboratory parameters, and urinary symptoms were recorded. Patients were stratified into two groups according to baseline NLR (<3 vs. ≥3). Urinary outcomes were assessed at baseline and 8 weeks using the International Prostate Symptom Score (IPSS) and the IPSS-related QoL item. Univariable and multivariable linear regression analyses were performed. **Results:** A total of 96 patients were analyzed. Median baseline NLR was 2.6 (IQR: 2.1–3.8). Patients with NLR ≥ 3 (*n* = 34) and NLR < 3 (*n* = 62) had comparable baseline characteristics and urinary scores. At 8 weeks, patients with NLR ≥ 3 experienced a greater worsening of urinary symptoms (median IPSS 24 vs. 21, *p* = 0.02; median change +5 vs. +2, *p* = 0.01) and QoL (median 5 vs. 4, *p* = 0.03). Univariable regression confirmed the association of NLR ≥ 3 with worse QoL (β = +0.74; *p* = 0.003) and higher IPSS (β = +2.20; *p* = 0.021). Modeled as a continuous variable, each one-unit increase in NLR corresponded to a +0.20 worsening in QoL (*p* = 0.008). In the multivariable analyses adjusted for baseline IPSS and concomitant CIS, NLR remained independently associated with QoL decline. **Conclusions:** Baseline NLR was independently associated with worsening urinary symptoms and QoL during BCG induction in NMIBC patients. NLR may represent a simple and accessible biomarker for early risk stratification during BCG induction, warranting validation in larger, longer-term prospective trials.

## 1. Introduction

The first-line treatment for patients with high-risk non-muscle-invasive bladder cancer (NMIBC) consists of transurethral resection of the bladder (TURB), followed by intravesical instillations of Bacillus Calmette–Guérin (BCG) [1]. Although BCG effectively reduces NMIBC recurrence and progression [2], approximately 70% of patients experience clinically relevant lower urinary tract symptoms (LUTS) during therapy, which negatively affect health-related quality of life (HRQoL) [3]. Moreover, 7–20% of patients discontinue treatment due to these adverse effects, thereby increasing the risk of disease progression and compromising survival [4,5].

Inflammation plays a pivotal role in mediating BCG’s therapeutic efficacy but also substantially contributes to BCG-induced urinary symptoms and adverse events [6]. Intravesical BCG instillation triggers a localized immune response characterized by the infiltration of macrophages, dendritic cells, and neutrophils into the bladder wall shortly after administration [7]. In addition, BCG-treated patients exhibit increased numbers of CD4+ and CD8+ T cells in urine and bladder mucosa [8]. Elevated levels of urinary cytokines, including IL-2, IL-6, IL-8, IL-18, IL-1ra, IFN-γ, IL-12[p70], TNF-α, and GM-CSF, as well as chemokines such as MCP-1, MIP-1α, and IP-10, have also been documented after BCG instillations [9,10].

The neutrophil-to-lymphocyte ratio (NLR) is an established marker of systemic inflammation in cancer patients, providing clinically relevant insights into disease status and treatment outcomes [11]. In bladder cancer, NLR has been associated with therapeutic efficacy and prognosis across several settings: in patients receiving neoadjuvant chemotherapy followed by cystectomy [12,13,14], in those with NMIBC at risk of recurrence [15], and in metastatic cases undergoing systemic palliative treatment. Yet, its potential association with BCG-related LUTS and, more importantly, with urinary symptom-related QoL has not been explored. Demonstrating such an association could provide clinicians with a simple, inexpensive, and widely available tool to identify patients at a higher risk of symptom burden and to implement early supportive strategies aimed at preserving treatment adherence and QoL.

We therefore hypothesized that elevated baseline systemic inflammation, as reflected by NLR, would be associated with worse urinary symptom-related QoL in NMIBC patients undergoing BCG therapy. The primary objective of this study was to evaluate the association between baseline NLR and urinary symptom-related QoL in this population. The secondary objective was to assess the association between baseline NLR and the overall severity of LUTS in the same population.

## 2. Materials and Methods

### 2.1. Study Design and Ethical Considerations

We designed a prospective, single-center, single-arm observational study, conducted at the University of Campania “Luigi Vanvitelli” (Naples, Italy) between January 2022 and June 2024. All enrolled patients received a standard induction course of intravesical BCG. For analytical purposes, baseline NLR was assessed both by stratifying patients into two groups (<3 vs. ≥3, according to predefined thresholds from the prior literature) and by modeling NLR as a continuous variable [16].

The study was approved by the local Ethics Committee (protocol n°261/2019) and conducted in accordance with the principles of the Declaration of Helsinki. Written informed consent was obtained from all participants for study participation and for the use of their data in scientific publications. Data were pseudonymized to ensure compliance with privacy regulations. Data processing complied with EU Regulation 2016/679 (GDPR) and applicable national legislation; all data were pseudonymized and stored on secure institutional servers within the EU.

### 2.2. Patient Population and Eligibility Criteria

We prospectively enrolled consecutive men diagnosed with NMIBC and scheduled to receive intravesical BCG following TURB. Eligibility required age ≥18 years, histologically confirmed high- or very high-risk NMIBC according to the 2025 EAU criteria [17], and no prior intravesical treatment.

To minimize the heterogeneity in LUTS assessment, the study population was limited to men, since male-specific factors such as prostate-related conditions, pharmacologic therapies targeting the prostate, and involvement of the prostatic urethra may significantly influence the urinary symptom burden. This restriction aimed to reduce measurement heterogeneity, since lower urinary tract symptoms in women may derive from different anatomical and functional factors, and the IPSS is validated primarily in male populations.

To control potential confounders of bladder inflammation and LUTS, patients were excluded if they had autoimmune or autoinflammatory disease, chronic use of anti-inflammatory drugs, daily use of tadalafil 5 mg, history of systemic chemotherapy or immunotherapy, hematologic disorders, recurrent or ongoing symptomatic urinary tract infection, chronic bacterial prostatitis, suspected or diagnosed prostate cancer, chronic pelvic pain syndrome, prior pelvic radiotherapy, previous bladder reconstruction, bladder stones, urethral stricture or prior urethral surgery, indwelling catheter, prior prostate surgery, neurogenic bladder dysfunction, uncontrolled diabetes mellitus, current use of sodium–glucose cotransporter-2 (SGLT2) inhibitors, or psychiatric/cognitive disorders. Patients with a known history or clinical suspicion of active or latent tuberculosis, or any other mycobacterial infection, were excluded in order to ensure safety with BCG therapy. Finally, men with missing data were excluded.

### 2.3. Treatment Details

All patients underwent TURB with complete resection, and the detrusor muscle was present in the specimen. Patients with T1 high-grade disease, incomplete resection, or suspected understaging were required to undergo a re-TURB within 2–6 weeks before starting BCG and were included only after adequate restaging had been achieved.

An immediate postoperative instillation of mitomycin C was administered in all patients. Subsequently, all subjects received the standard induction course of six weekly intravesical BCG instillations [17]. Patients who did not receive mitomycin C or who failed to complete the full induction cycle for any reason were excluded from the analysis.

All resections were performed by attending urologists. All histopathological specimens were reviewed by a dedicated genitourinary pathologist, and instillations were administered according to a standardized institutional protocol.

The analysis was deliberately restricted to the induction phase, as this represents the most critical treatment window for the onset of urinary symptoms and the risk of early treatment discontinuation. Moreover, this approach allowed us to avoid confounding related to heterogeneous maintenance schedules.

The use of medications with potential impact on urinary symptoms/discomfort or inflammatory response (e.g., non-steroidal anti-inflammatory drugs (NSAIDs), corticosteroids, nutraceuticals, antibiotics, analgesics, and antispasmodics) during the induction phase was discouraged unless clinically necessary. Chronic medications for benign prostatic hyperplasia (BPH)-related LUTS were permitted, provided they had been stable for at least 3 months prior to enrollment.

### 2.4. Patient Assessment and Study Endpoints

Baseline assessment, including clinical evaluation and laboratory testing, was performed at the visit immediately prior to the first intravesical instillation, after TURB and adequate recovery. Baseline hematology parameters were obtained from peripheral venous blood at the pre-instillation baseline visit (after TURB) as part of a complete blood count. NLR was calculated as the ratio of absolute neutrophil count to absolute lymphocyte count from the same sample. Because baseline assessments were performed after TURB and before BCG induction, residual influence from surgical recovery cannot be excluded.

The baseline variables recorded included age, body mass index (BMI; Kg/m^2^), absolute neutrophil count, absolute lymphocyte count, smoking status, major comorbidities, NLR (recorded as a continuous variable and dichotomized as <3 vs. ≥3), hemoglobin (Hb; g/dL), platelets (PLT; ×10^9^/L), tumor stage, tumor grade, presence of concomitant carcinoma in situ (CIS), and current use of BPH medications (e.g., α-blockers, 5-α-reductase inhibitors). Their distribution was balanced across groups and therefore not included as adjustment variables in the multivariable models. At baseline, subjects completed the International Prostate Symptom Score (IPSS), including the quality-of-life (QoL) item [18]. At 8 weeks, immediately after completion of the six-week induction course, urinary symptoms were reassessed using both the IPSS total score and the QoL item.

The primary endpoint was the association between baseline NLR and the 8-week IPSS-QoL item, reflecting urinary symptom-related QoL. The secondary endpoint was the association between baseline NLR and the 8-week IPSS total score. Exploratory analyses evaluated the associations between NLR and baseline clinico-pathological or treatment-related variables.

### 2.5. Statistical Analysis

The sample size was calculated based on the primary endpoint (IPSS-QoL at 8 weeks). A 1-point difference on the IPSS-QoL item was selected as the clinically relevant threshold, consistent with prior reports indicating a minimum clinically important difference (MCID) of approximately 0.5 points [19]. A standard deviation (SD) of 1.5 and a 65:35 group allocation (NLR < 3:NLR ≥ 3) were assumed according to the available literature [16]. Under these conditions, with a two-sided α = 0.05 and 80% power, the required sample size was estimated at 78 patients. To account for the use of non-parametric testing and potential attrition, a conservative 10% inflation was applied, resulting in a final target of 86 patients (≈56 and 30 per group)

Continuous variables were summarized as mean ± or median with interquartile range (IQR), according to the distribution assessed by the Shapiro–Wilk test. Categorical variables were reported as absolute numbers and percentages. Between-group comparisons (NLR < 3 vs. ≥3) were performed using the unpaired Student’s t-test or Mann–Whitney U test for continuous variables, and the Chi-squared or Fisher’s exact test for categorical variables, as appropriate.

For the primary endpoint (IPSS-QoL at 8 weeks) and the secondary endpoint (IPSS total score at 8 weeks), univariable and multivariable linear regression models were fitted, with IPSS outcomes as dependent variables and baseline NLR (binary or continuous) as the independent variable. Results were reported as β coefficients with 95% confidence intervals (CI). To facilitate interpretation, regression results for IPSS outcomes were graphically displayed in forest plots, illustrating β coefficients with 95% CIs. Multivariable models were adjusted a priori for the baseline IPSS (to account for the initial symptom burden) and the presence of concomitant CIS, selected on clinical plausibility. To limit overfitting given the sample size, only these prespecified covariates were retained in the main models. Additional factors (e.g., BPH medications, smoking status, major comorbidities) were examined in sensitivity analyses.

Exploratory analyses assessed the associations between baseline NLR and clinico-pathological or treatment-related characteristics, using linear or logistic regression as appropriate.

All statistical tests were two-tailed, with *p* < 0.05 considered statistically significant. Analyses were performed using R v.4.5.1 (R Foundation for Statistical Computing, Vienna, Austria) within the RStudio v.2025.05.1+513 environment (Posit, Boston, MA, USA).

## 3. Results

During the study period, 114 patients were assessed for eligibility. After applying prespecified inclusion/exclusion criteria, 104 patients-initiated BCG induction. Of these, 96 (92.3%) completed the full six-week course and were included in the final analysis. Patients who did not complete induction (*n* = 8, 7.7%) were excluded for the following reasons: adverse events (*n* = 4; severe cystitis or febrile urinary tract infection), patient refusal (*n* = 2; treatment-related discomfort), or protocol deviation (*n* = 2; incomplete outcome assessment). The baseline characteristics are summarized in Table 1. The median baseline NLR was 2.6 (IQR: 2.1–3.8), with a clear separation between patients in the NLR < 3 group (*n* = 62; median 1.9, IQR: 1.6–2.2) and those in the NLR ≥ 3 group (*n* = 34; median 4.1, IQR: 3.5–4.8; *p* < 0.001). No significant differences between groups were observed for age, BMI, smoking status, major comorbidities, hemoglobin, platelet count, or concomitant CIS. Baseline urinary symptom burden was comparable, with median IPSS total scores of 19 (IQR: 16–21) in the NLR < 3 group and 18 (IQR: 17–23) in the NLR ≥ 3 group (*p* = 0.06). Similarly, the baseline IPSS-QoL did not differ significantly (median 3 vs. 4; *p* = 0.09).

**Figure 1:**
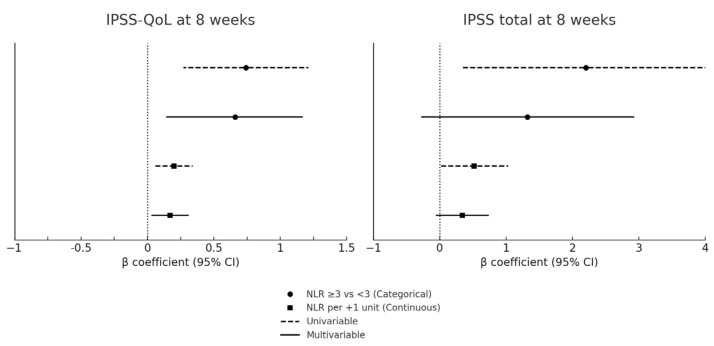
Forest plot of baseline NLR and IPSS outcomes at 8 weeks. CI: confidence interval; CIS: carcinoma in situ; IPSS: International Prostate Symptom Score; NLR: neutrophil-to-lymphocyte ratio; QoL: quality of life. Forest plots of univariable and multivariable regression models assessing the association between baseline NLR and IPSS outcomes at 8 weeks. The y-axis shows the predictors (categorical NLR: ≥3 vs <3; continuous NLR: per 1-unit increase), and the x-axis displays β coefficients with 95% confidence intervals. Circles represent categorical analyses and squares continuous analyses. Dashed lines denote univariable models, while solid lines denote multivariable models adjusted for baseline IPSS score and concomitant CIS. The vertical dashed line indicates the null effect (β = 0). Positive β values correspond to worse urinary symptoms or poorer QoL. Numerical estimates are reported in Table 3.

At 8 weeks, following completion of the induction course, the patients with baseline NLR ≥ 3 reported significantly worse urinary outcomes compared with those with NLR < 3. The median IPSS total score increased to 24 (IQR: 21–27) in the NLR ≥ 3 group vs. 21 (IQR: 18–23) in the NLR < 3 group (*p* = 0.02), corresponding to a median change of +5 points (IQR: 3–7) vs. +2 points (IQR: 1–3; *p* = 0.01). Urinary symptom–related QoL also deteriorated more markedly in the NLR ≥ 3 group, with an 8-week median IPSS-QoL of 5 (IQR: 4–6) compared with 4 (IQR: 3–5) in the NLR < 3 group (*p* = 0.03). The absolute change in QoL score was significantly greater in patients with NLR ≥ 3 (+1 [IQR: 1–2] vs. +1 [IQR: 0–1]; *p* = 0.04). IPSS outcomes at baseline and 8 weeks according to NLR group are reported in Table 2. Baseline NLR remained independently associated with higher IPSS-QoL scores at 8 weeks. A univariable regression confirmed that patients with baseline NLR ≥ 3 had significantly worse urinary outcomes at 8 weeks, with higher IPSS-QoL scores (β = +0.74, 95% CI: 0.27–1.21; *p* = 0.003) and higher IPSS total scores (β = +2.20, 95% CI: 0.35–4.05; *p* = 0.021) (Table 3). When NLR was analyzed as a continuous variable, each 1-unit increase was associated with a 0.20-point worsening in IPSS-QoL (95% CI: 0.05–0.34; *p* = 0.008) and a 0.52-point increase in IPSS total score (95% CI: 0.02–1.03; *p* = 0.042). In multivariable models adjusted for baseline IPSS and concomitant CIS, NLR remained an independent predictor of QoL deterioration (β = +0.66, 95% CI: 0.14–1.17; *p* = 0.014; per 1-unit increase: β = +0.17, 95% CI: 0.03–0.31; *p* = 0.019), whereas its association with IPSS total score was attenuated and no longer statistically significant. The results of the regression analyses are detailed in Table 3 and illustrated in Figure 1.

Sensitivity analyses adjusting for potential confounders, including BPH medications, smoking status, and major comorbidities, confirmed the robustness of these associations, with regression coefficients remaining consistent in both direction and magnitude. Exploratory analyses showed no significant relationships between baseline NLR and tumor stage (Ta vs. T1; *p* = 0.14), tumor grade (*p* = 0.21), or concomitant CIS (*p* = 0.07).

The between-group difference in IPSS-QoL at 8 weeks was approximately 1 point (median 5 vs. 4; NLR ≥ 3 vs. NLR < 3), exceeding the commonly cited MCID of 0.5 points for this item.

## 4. Discussion

### 4.1. Methodological Considerations

The IPSS and its QoL item have been widely validated as symptom assessment tools in patients with lower urinary tract symptoms, including those with NMIBC [20]. While this instrument is male-oriented, its consistent use in NMIBC research makes it a pragmatic choice in this setting. Nonetheless, comparative studies have demonstrated the value of alternative validated instruments such as the Bristol Female Lower Urinary Tract Symptoms questionnaire (BFLUTS) and the Overactive Bladder questionnaire (OAB-q), particularly in female cohorts [21]. Our findings should therefore be interpreted within this methodological framework, and future studies would benefit from integrating multiple validated QoL instruments.

### 4.2. Main Findings and Available Evidence

In this prospective, single-center cohort of male patients with high- or very-high-risk NMIBC undergoing a uniform BCG induction, a higher baseline NLR was associated with an early deterioration in urinary symptom–related QoL at 8 weeks. Using a prespecified threshold, patients with NLR ≥ 3 reported worse IPSS-related QoL than those with NLR < 3, and this association persisted after adjustment for baseline symptom burden and concomitant CIS. When modeled continuously, a higher NLR remained associated with poorer QoL, whereas the association with the total IPSS attenuated in multivariable analyses.

Overall, these data suggest that baseline systemic inflammation, proxied by NLR, is associated with early patient-reported urinary burden during BCG induction. NLR emerged as independently associated with QoL outcomes during BCG induction.

These findings are concordant with the established symptomatic profile of BCG. Prior studies document that BCG frequently induces clinically relevant lower urinary tract symptoms and negatively impacts health-related QoL, with cystitis-like symptoms and treatment discontinuations most pronounced around the induction window [22,23].

Our findings are in line with previous reports demonstrating that intravesical BCG is associated with a temporary but clinically meaningful deterioration of urinary function and QoL during the induction phase. In the EORTC trial by Sylvester et al. [24] patients reported a high rate of urinary frequency and dysuria during the first 6 weeks of therapy, which subsequently improved once induction ended. Similarly, Oddens et al. [25] documented that the impact on QoL is most pronounced during induction, with stabilization over maintenance cycles. Our observation of a significant QoL decline at 8 weeks, followed by stabilization, is consistent with these temporal patterns.

A recent synthesis of mechanisms and management strategies further supports a pathophysiological link between BCG-mediated immune activation and LUTS, reinforcing the biological plausibility of our observations [14]. Moreover, comparative data indicates that QoL metrics often favor intravesical chemotherapy over BCG in selected settings, underlining the higher symptomatic burden associated with BCG-based immunotherapy [26]. With respect to systemic inflammatory markers, the current evidence base in bladder cancer has primarily linked NLR to oncologic endpoints rather than treatment-related urinary toxicity. Elevated NLR has been associated with adverse pathology and survival in muscle-invasive disease, including patients treated with neoadjuvant chemotherapy and radical cystectomy [22,23], and with recurrence/progression risks in non–muscle-invasive cohorts, as summarized in primary studies and meta-analyses [9,27].

Templeton et al. [28] conducted a large meta-analysis showing that elevated NLR is associated with worse survival across multiple tumor types, including bladder cancer. While our study does not address survival, it extends this concept to patient-reported outcomes, showing that baseline NLR is independently associated with short-term QoL decline during BCG induction. From a clinical perspective, these findings underline the importance of considering systemic immune status when counseling patients before BCG therapy. Identifying individuals at risk of QoL deterioration may enable timely supportive measures, such as optimized management of urinary symptoms, to improve adherence and reduce the risk of treatment discontinuation.

Our data complement the literature by focusing on a distinct, patient-centered outcome—urinary symptom–related QoL—during BCG induction, an area for which specific evidence remains limited. The biological rationale is supported by experimental and translational observations showing early neutrophil recruitment and heightened cytokine milieu after intravesical BCG, which may amplify local symptoms [15,16,17]. Other inflammatory indices have also shown prognostic value for oncologic outcomes in urothelial carcinoma, such as the systemic immune-inflammation index (SII), modified Glasgow prognostic score (mGPS), and platelet/lymphocyte-derived ratios, across cystectomy and NMIBC settings [29,30]. While these markers underscore the broad relevance of systemic inflammation in bladder cancer, the evidence directly connecting them to BCG-related urinary toxicity is scarce. Taken together, the available data support a consistent role for systemic inflammation in bladder cancer biology, and our findings extend this concept to early, clinically meaningful, patient-reported outcomes under BCG.

### 4.3. Strengths and Limitations

To our knowledge, this is the first prospective study to link baseline NLR with patient-reported urinary outcomes under BCG therapy. In addition, this research has several strengths. It was prospectively designed, with standardized data collection and clearly prespecified endpoints, which minimizes recall bias. Patients were stratified into two groups according to baseline NLR, a widely adopted and clinically meaningful threshold, thereby facilitating the interpretability of the findings. The cohort was homogeneous, including only male patients with high- or very high-risk NMIBC receiving a uniform induction regimen of BCG, which reduced the heterogeneity related to sex-specific differences and treatment schedules. Pathological evaluation by a single dedicated genitourinary pathologist, transurethral resections performed by experienced urologists, and intravesical instillations administered by experienced oncologists ensured internal consistency across diagnostic and therapeutic steps. Moreover, the use of validated patient-reported outcome instruments (IPSS and the IPSS-related QoL item) strengthened the reliability and clinical relevance of the functional and QoL assessments.

Nonetheless, several limitations should be acknowledged. First, as this was an observational study, no causal inference can be drawn. The study was conducted at a single institution, which may limit generalizability. Only male patients were included, which enhances internal validity but precludes extrapolation to female populations. Second, although the use of medications potentially affecting LUTS or inflammation during the induction phase was discouraged, deviations from this recommendation were not systematically recorded, and their impact cannot be fully excluded. Third, the study did not incorporate additional inflammatory markers such as C-reactive protein or other systemic indices, which could have provided complementary information on systemic inflammation. Fourth, baseline IPSS and QoL were assessed after TURB. Therefore, post-surgical effects cannot be fully disentangled from treatment-related changes. However, baseline scores were comparable across groups, which mitigates this potential bias. The observed difference in IPSS-QoL (~1 point) exceeds the published. Fifth, oncological outcomes were not evaluated, since the study was deliberately focused on functional and inflammatory endpoints within the induction phase. In addition, follow-up was limited to the induction phase, and longer-term outcomes (e.g., at 6 or 12 months) were not collected. Finally, although multivariable adjustments were applied, the possibility of residual confounding from unmeasured covariates cannot be entirely ruled out, and causality cannot be inferred from this observational design.

### 4.4. Future Perspectives

Future research should validate these findings in larger, multicenter cohorts to strengthen external validity. Importantly, subsequent investigations should also include female patients, as sex-related differences in bladder physiology, immune response, and treatment-related toxicity may influence the association between NLR and BCG-related urinary outcomes. Extending the observation period to maintenance therapy and long-term follow-up would clarify whether baseline NLR predicts sustained urinary symptom trajectories, adherence to BCG, and ultimately oncological outcomes.

The incorporation of additional inflammatory and immunological markers, such as C-reactive protein, composite immune–inflammation indices, or urinary cytokine profiles, may further elucidate the biological mechanisms linking baseline systemic inflammation to BCG-induced urinary toxicity. Although NLR is inherently a blood-derived index, other immune-related biomarkers in urine or tumor tissue (e.g., cytokines, tumor-infiltrating lymphocytes) may provide complementary information, but these were beyond the scope of the present analysis. Moreover, future studies should investigate whether NLR can be integrated with clinical, pathological, and molecular variables into comprehensive predictive models for BCG-related adverse events. Such models could enable an early identification of patients at increased risk, thereby facilitating the implementation of tailored strategies to optimize adherence and preserve QoL. Future studies should include female cohorts assessed with validated instruments such as the Bristol Female Lower Urinary Tract Symptoms questionnaire (BFLUTS) or the Overactive Bladder questionnaire (OAB-q) to broaden external validity. Finally, interventional studies testing pharmacological and non-pharmacological approaches to mitigate urinary symptoms in patients with elevated baseline NLR may represent an important step toward a more personalized and tolerable management of NMIBC during BCG therapy.

Future studies will also include 6- and 12-month follow-ups to assess whether baseline NLR predicts sustained QoL trajectories and adherence over time.

## 5. Conclusions

Baseline NLR was independently associated with a short-term worsening of urinary symptoms and QoL during BCG induction in NMIBC patients. While these findings suggest a potential role for NLR in early risk stratification, they should be interpreted with caution given the study limitations. Future well-designed trials are needed to confirm its predictive value and to clarify how this marker could be integrated into patient management and supportive strategies aimed at preserving treatment adherence and patient well-being.

## Figures and Tables

**Table 1:** Baseline characteristics of the study population according to NLR group (<3 vs. ≥3).

Parameter	Total (*n* = 96)	NLR < 3 (*n* = 62)	NLR ≥ 3 (*n* = 34)	*p*-Value
Age, years *Median [IQR]*	71 [68–75]	72 [69–76]	70 [67–73]	0.07
BMI, kg/m^2^ *Median [IQR]*	26.3 [24.1–28.8]	26.1 [24.0–28.5]	26.8 [24.3–29.2]	0.29
Smoking, *n* (%)	19 (19.8)	11 (17.7)	8 (23.5)	0.49
Hypertension, *n* (%)	56 (58.3)	36 (58.1)	20 (58.8)	0.94
Diabetes, *n* (%)	18 (18.8)	11 (17.7)	7 (20.6)	0.72
BPH medications, *n* (%)	71 (74.0)	44 (71.0)	27 (79.4)	0.36
Hb levels, g/dL *Mean (SD)*	11.9 (1.7)	12.0 (1.6)	11.7 (1.8)	0.38
PLT, ×10^9^/L *Median [IQR]*	259 [241–279]	262 [245–281]	254 [236–275]	0.08
NLR *Median [IQR]*	2.6 [2.1–3.8]	1.9 [1.6–2.2]	4.1 [3.5–4.8]	<0.001
Tumor stage, *n* (%) Ta T1	27 (28.1) 69 (71.9)	17 (27.4) 45 (72.6)	10 (29.4) 24 (70.6)	0.84
Tumor grade, *n* (%) High Low	96 (100) 0	62 (100) 0	34 (100) 0	NA
Concomitant CIS *, *n* (%)	29 (30.2)	18 (29.0)	11 (32.4)	0.72
IPSS total, points *Median [IQR]*	19 [17–22]	19 [16–21]	18 [17–23]	0.06
IPSS-QoL, points *Median [IQR]*	4 [3–4]	3 [3–4]	4 [3–5]	0.09

BMI: body mass index; BPH: benign prostatic hyperplasia; CIS: carcinoma in situ; Hb: hemoglobin; IQR: interquartile range; IPSS: International Prostate Symptom Score; NA: not available; NLR: neutrophil-to-lymphocyte ratio; QoL: quality of life; SD: standard deviation. * No cases of isolated CIS (Tis) were recorded. Continuous variables are reported as mean (SD) or median [IQR], as appropriate; categorical variables are reported as *n* (%). *p*-values were calculated using unpaired t-test/Mann–Whitney U test (continuous) and Chi-squared/Fisher’s exact test (categorical). Bold *p*-values indicate statistical significance (*p* < 0.05).

**Table 2:** IPSS outcomes at baseline and 8 weeks according to NLR group (<3 vs. ≥3).

Outcome	Total (*n* = 96)	NLR < 3 (*n* = 62)	NLR ≥ 3 (*n* = 34)	*p*-Value
IPSS total (baseline), *Median [IQR]*	19 [17–22]	19 [16–21]	18 [17–23]	0.06
IPSS total (8 weeks), *Median [IQR]*	22 [19–25]	21 [18–23]	24 [21–27]	0.02
Δtotal (8w—baseline), *Median [IQR]*	+3 [2–5]	+2 [1–3]	+5 [3–7]	0.01
IPSS-QoL (baseline), *Median [IQR]*	4 [3–4]	3 [3–4]	4 [3–5]	0.09
IPSS-QoL (8 weeks), *Median [IQR]*	5 [4–6]	4 [3–5]	5 [4–6]	0.03
ΔQoL (8w—baseline), *Median [IQR]*	+1 [0–2]	+1 [0–1]	+1 [1–2]	0.04

IPSS: International Prostate Symptom Score; IQR: interquartile range; NLR: neutrophil-to-lymphocyte ratio; QoL: quality of life. Continuous variables are reported as median [IQR]. Δ indicates the absolute change from baseline to 8 weeks. *p*-values refer to between-group comparisons (NLR < 3 vs. NLR ≥3) and were calculated using the Mann–Whitney U test. Bold *p*-values indicate statistical significance (*p* < 0.05).

**Table 3:** Univariable and multivariable regression models for the association between baseline NLR and IPSS outcomes at 8 weeks.

Outcome	Model	Predictor	β (95% CI)	*p*-Value
IPSS-QoL (primary)	Univariable	NLR ≥ 3 (vs. <3)	+0.74 (0.27 to 1.21)	0.003
	Univariable	NLR (per 1-unit increase)	+0.20 (0.05 to 0.34)	0.008
	Multivariable ^1^	NLR ≥ 3 (vs. <3)	+0.66 (0.14 to 1.17)	0.014
	Multivariable ^1^	NLR (per 1-unit increase)	+0.17 (0.03 to 0.31)	0.019
IPSS total (secondary)	Univariable	NLR ≥ 3 (vs. <3)	+2.20 (0.35 to 4.05)	0.021
	Univariable	NLR (per 1-unit increase)	+0.52 (0.02 to 1.03)	0.042
	Multivariable ^2^	NLR ≥ 3 (vs. <3)	+1.32 (−0.28 to 2.93)	0.104
	Multivariable ^2^	NLR (per 1-unit increase)	+0.34 (−0.06 to 0.74)	0.096

CI: confidence interval; CIS: carcinoma in situ; IPSS: International Prostate Symptom Score; NLR: neutrophil-to-lymphocyte ratio; QoL: quality of life. Linear regression analyses were performed. β coefficients represent the change in the 8-week outcome associated with NLR. Univariable models included NLR only. ^1^ Multivariable models adjusted a priori for baseline QoL and concomitant CIS (yes/no); ^2^ Multivariable models adjusted a priori for baseline IPSS total and concomitant CIS (yes/no). Bold *p*-values indicate statistical significance (*p* < 0.05).

## Data Availability

Raw data are available upon justified request to the corresponding author.
